# Building Heating and Cooling Load Prediction Using Ensemble Machine Learning Model

**DOI:** 10.3390/s22197692

**Published:** 2022-10-10

**Authors:** Rajasekhar Chaganti, Furqan Rustam, Talal Daghriri, Isabel de la Torre Díez, Juan Luis Vidal Mazón, Carmen Lili Rodríguez, Imran Ashraf

**Affiliations:** 1 Toyota Research Institute, Los Altos, CA 94022, USA; 2 School of Computer Science, University College Dublin, D04 V1W8 Dublin, Ireland; 3 Department of Industrial Engineering, Jazan University, Jazan 45142, Saudi Arabia; 4 Department of Signal Theory and Communications and Telematic Engineering, University of Valladolid, Paseo de Belén 15, 47011 Valladolid, Spain; 5 Universidad Europea del Atlántico, Isabel Torres 21, 39011 Santander, Spain; 6 Universidad Internacional Iberoamericana Arecibo, Arecibo, PR 00613, USA; 7 Universidade Internacional do Cuanza, Cuito P.O. Box 841, Bié, Angola; 8 Universidad Internacional Iberoamericana, Campeche 24560, Mexico; 9 Department of Information and Communication Engineering, Yeungnam University, Gyeongsan 38541, Korea

**Keywords:** energy consumption prediction, cooling load, smart homes, Internet of Things, sustainable homes

## Abstract

Building energy consumption prediction has become an important research problem within the context of sustainable homes and smart cities. Data-driven approaches have been regarded as the most suitable for integration into smart houses. With the wide deployment of IoT sensors, the data generated from these sensors can be used for modeling and forecasting energy consumption patterns. Existing studies lag in prediction accuracy and various attributes of buildings are not very well studied. This study follows a data-driven approach in this regard. The novelty of the paper lies in the fact that an ensemble model is proposed, which provides higher performance regarding cooling and heating load prediction. Moreover, the influence of different features on heating and cooling load is investigated. Experiments are performed by considering different features such as glazing area, orientation, height, relative compactness, roof area, surface area, and wall area. Results indicate that relative compactness, surface area, and wall area play a significant role in selecting the appropriate cooling and heating load for a building. The proposed model achieves 0.999 $R^{2}$ for heating load prediction and 0.997 $R^{2}$ for cooling load prediction, which is superior to existing state-of-the-art models. The precise prediction of heating and cooling load, can help engineers design energy-efficient buildings, especially in the context of future smart homes.

## 1. Introduction

Internet of Things (IoT) is revolutionizing many sectors to improve productivity and advance the human lifestyle. The smart home is one such example of IoT applications to enhance energy efficiency and sustainability [1]. Several physical devices are connected to the Internet for monitoring and tracking the status of the physical environment and house activities. The human being does not need to be at home to perform these activities. As billions of IoT devices need power to operate, energy resources are scarce, and preserving energy resources is very important. For estimating the cooling or heating load of a building, temperature characteristics or profiles of smart homes should be analyzed. If the relation between the building structure and energy requirements is known, then the builder designs and architects the building architecture, which can save energy and effectively use the energy to heat or cool the building. Therefore, the heating load (HL) and cooling load (CL) of a building estimation have been a problem in the building energy efficiency field.

Energy consumption prediction is an important research area as it consumes approximately 30% of the total energy and its carbon emissions are approximately 33% in 2021 [2]. Despite developments in the building sector, the current efforts are not enough to achieve the 1.5 °C scenario. Smart sustainable infrastructures are needed to meet the rapidly growing urbanization. From this perspective, energy consumption prediction and modeling is an important task to obtain smart and low energy-requiring infrastructures. Three primary approaches for building energy consumption modeling and forecasting are physical models, data-driven models, and hybrid models [3]. Data-driven approaches have been regarded as the most suitable for integration into smart houses.

With the wide deployment of IoT sensors in smart homes and appliances, such sensors generate large amounts of data. Data-driven approaches leverage the data for modeling and forecasting energy consumption patterns. Furthermore, the use of machine learning and deep learning models provide flexible and reliable solutions in this regard [4]. Several solutions exist for building energy prediction in the literature [5,6,7]. However, such solutions lack several aspects. First, predominantly, studies focus on the use of individual machine learning models, and ensemble models are not properly investigated. Second, the performance of such models still needs improvement regarding prediction accuracy. Third, often the models are optimized by fine-tuning different hyperparameters, and the potential of feature selection is partially investigated. In addition, the role of different attributes such as roof area, wall area, glazing, etc. is not very well studied. As a result, such studies are not very well suited for predicting the energy needs of modern smart homes. This study aims to overcome these issues.

This study follows a machine learning-based data-driven approach for building energy consumption prediction and makes the following contributions:

- A novel ensemble model is proposed that combines three random forest models (3RF) for predicting the heating and cooling load of the buildings. Performance comparison of the proposed approach is carried out with reference to K-nearest neighbor (KNN), linear regression (LR), random forest (RF), general additive model (GAM), and multilayer perceptron (MLP). In addition, convolutional neural networks (CNN), long short-term memory (LSTM), and their ensemble CNN-LSTM are also used for experiments.
- The influence of different features related to building is investigated. The performance of the models is analyzed regarding different features from the dataset such as glazing area, orientation, height, relative compactness, roof area, surface area, and wall area. Mean absolute error, root mean squared error, mean absolute percentage error, and coefficient of determination R-squared is used for performance evaluation.
- Performance is also evaluated within the context of existing state-of-the-art studies regarding prediction accuracy and computational complexity.

The rest of the article is organized as follows. Section 2 includes state-of-the-art works using machine learning and deep learning models to predict the building energy efficiency performance. Section 3 discusses the proposed methodology to predict heat loading and cool loading in buildings. Section 3.3 covers the description of the building energy efficiency simulated datasets. Section 4 discusses the performance results of the proposed machine learning and deep learning models to predict heat and cool loading. Section 5 concludes the article.

## 2. Literature Review

Energy usage optimization and energy saving of the buildings are needed to conserve energy. Statistical modeling techniques are commonly used to predict the energy consumption of the building given the building characteristics. Researchers applied various machine learning models to predict energy consumption and efficiency. The building energy efficiency is measured in terms of heat loading and cool loading parameters.

Kumar et al. [5] proposed extreme learning machine (ELM) and online sequential ELM (OSELM) methods to predict the building HL and CL parameters. The combinations of ELM methods are used to compare the prediction performance of the models with well-known ML models such as artificial neural network (ANN), support vector machine (SVM), gradient boosting (GB), etc. The authors also varied the attribute combinations to compare the performance of the proposed methods. The reported results indicate that ELM with group 4 attribute combination produced the best result with a 0.0456 mean absolute error (MAE) for HL and 0.0358 for CL prediction. An ANN-based solution is proposed in [6] to predict the cooling and heating load in the buildings. A component-based approach is taken to predict the energy efficiency of the buildings. The ANN is applied at each component level to obtain the final HL and CL performance results. The Energyplus software is used to generate the simulation-based datasets. The reported results show that $R^{2}$ is 0.974 for HL and 0.999 for CL. The proposed solution is a component-based approach with the advantage of the reusability of the ML models. However, the ML model may suffer from data or concept drift.

The authors presented an LSTM-based deep learning solution to predict the HL and CL performance in [8]. Transfer learning and multitask-based learning are also embedded in the solution to predict building energy efficiency. One of the advantages of deep learning models is the computation speed compared to building performance simulation (BPS). The method obtained an $R^{2}$ of 0.983 for CL and 0.848 for HL performance. The HL performance still requires improvement. Additionally, the training time for LSTM-based solutions is much higher than the ANN and machine learning-based solutions. XGboosting algorithm is proposed in [9] to predict the building energy load in terms of HL and CL. The performance metric’s root mean squared error (RMSE), $R^{2}$, MAE, and mean absolute percentage error (MAPE) are measured for both the HL and CL prediction. The reported results show that the obtained $R^{2}$ for HL is 0.9993, while CL has a 0.998 $R^{2}$. Chakraborty et al. [10] performed an in-depth study of feature selection, feature engineering, and parameter optimization to predict the building energy loads on the generated synthetic dataset. Machine learning and deep learning techniques such as XGBoost and ANN are used to conduct the experiments and predict the HL and CL. Results indicate that the XGBoost obtained RN_RMSE of 2.95 for CL and 3.90 for HL, while the $R^{2}$ value of 0.98 for CL and 0.95 for HL are obtained when the feature engineering is performed. However, the feature selection and hyperparameter optimization are not included. The authors pointed out that the scarcity of datasets hinders the research progress in this context. Octahedral regression-based building energy load prediction is proposed in [7]. Octahedral regression is a kernel averaging methodology proposed by authors to predict the building energy loads. Experimental results indicate an MAE value of 0.945 for HL, whereas the MAE for CL is 1.113. The prediction performance still has room for improvement.

The authors propose a multi-objective optimization model in [11] to predict the building energy load. The method MOO was also presented to improve the hyperparameter selection process. The authors claim that the proposed optimized model enhances the energy prediction accuracy and computation time compared to the grid search model parameter selection. The experimental results on simulated building datasets show that the HL and CL obtained higher $R^{2}$ values of 0.992 and 0.993, respectively. Sadeghi et al. [12] implemented a deep neural network (DNN) model to predict the building energy load prediction. The authors show that the DNN performed better than ANN to estimate the energy load. Normalized RMASE (NRMSE) and normalized MAE (NMAE) are used for evaluation. The $R^{2}$ value is 1 for the HL case, whereas 0.994 for the CL case. However, the training time for DNN is higher than for machine learning models. Song et al. [13] proposed a temporal convolution neural network (TCN) to predict hourly heat loading. The TCN has the advantage of rapidly extracting complex features due to the presence of a convolution neural network (CNN) and recursive neural network (RNN). The hourly heat loading performance accuracy is reported as 97.9% and an MAE value of 0.102 on the test dataset. However, the applicability of the TCN algorithm in building energy load prediction is not explored in the study.

Sajjad et al. [14] presented a sequential learning model framework to predict the HL and CL as multi-outputs. The authors claimed that their work is the first to propose a multi-output HL and CL prediction using a gated recurrent unit (GRU) to eliminate the tedious task of training separately for HL and CL prediction. The HL obtained a MAPE of 0.9315, and CL obtained a MAPE of 1.0132. However, the training time for the neural networks is much higher than for the machine learning models. The training time-based comparison is not discussed in the article. A feature construction method along with ensemble learning algorithms is proposed to predict the CL prediction in [15]. Different methods, such as K-clustering, discrete wavelet transform (DWT), etc., were used for feature construction, and three models of gradient boosting machine (GBM), random forest (RF), and cubist algorithms were used to evaluate the cooling load accuracy. The dataset was obtained from the Tianjin office building in China. The reported results indicate that the CL achieved an $R^{2}$ of 99.8% when the combination of DWT and the Cubist algorithm was used for evaluation. However, this work is limited to cooling load prediction.

Olu et al. [16] performed building energy performance prediction at the building design stages. The authors explored various machine learning techniques to evaluate energy performance. The feature selection and hyperparameter selection were also considered to improve the performance. The GB performed better than all other machine learning models with an accuracy of 67% and an F1 score of 65%. A shuffled complex evolution (SCE) performance optimization technique is proposed in [17] to enhance the performance of multi-layer perceptron (MLP). The authors claim that SCE improved the prediction accuracy by 22.84% and outperformed the benchmark optimizer such as moth-flame and optics-inspired models. The CL prediction results show that the SCE_MLP model achieved a 0.9227 $R^{2}$ for the well-known building energy efficiency datasets [18].

Table 1 provides an analytical overview of the state-of-the-art works, which worked on progressively improving the building energy efficiency. Several works in the literature can be found that have worked on improving the performance of HL and CL in solving the building energy efficiency problem. Although the reported results showed significant prediction performance, there is still scope for improvement. Additionally, the detailed study of the input features and output is not well explored in the literature. The prior works do not address an ideal solution that improves the performance and reduces the computation and training time. Thus, a detailed study on the building energy datasets is performed using machine learning and deep learning techniques, feature selection, and hyperparameter tuning to obtain an accurate, generalized, and low training time solution.

## 3. Proposed Methodology

**Figure 1:**
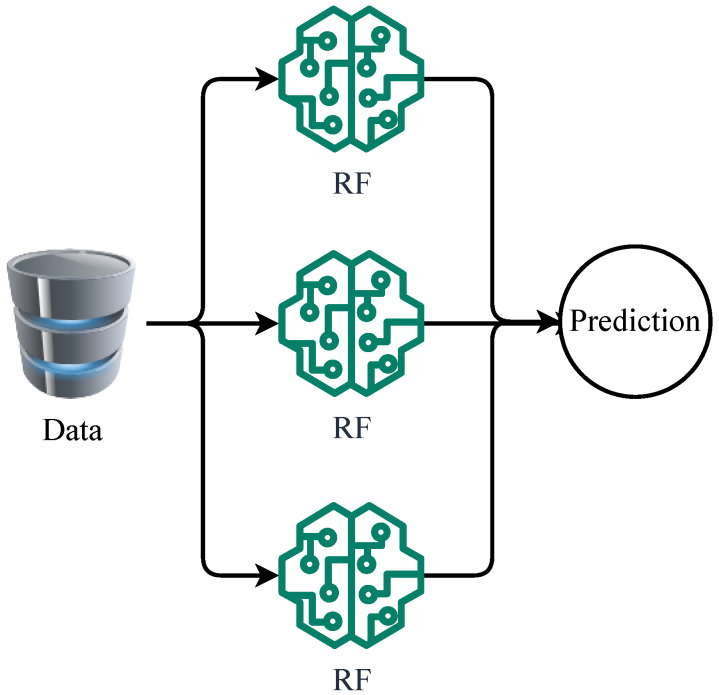
The architecture of the proposed 3RFF model HL and DL prediction.

Our motivation for this study is to leverage the data analytic models for predicting energy resource consumption in buildings. To improve the prediction performance of the state-of-the-art energy efficiency HL and CL, the selected machine learning models are thoroughly evaluated. This study proposes an ensemble model 3RandomForest (3RF) to predict the HL and CL of a building effectively. The architecture of the proposed 3RF model is presented in Figure 1. Three RF models are used to test the dataset and predict the HL and CL. The advantage of our method is to leverage the combinations of more decision trees from three RF models and achieve optimal performance results.

### 3.1. 3Random Forest

The decision tree models showed promising results for predicting the HL and DL. In order to improve the performance, 3RF is proposed to repeat the RF model regression prediction three times on the energy efficiency dataset. This repetition helps to use more combinations of the input dataset decision trees. The voting method is used to predict the final value from three RF models.

Let b={1,2,…,B} be the number of decision trees. ${\hat{C}}_{b}\left(x\right)$ denotes the regression prediction value of the $b^{th}$ decision tree [19]. Then, the output of the RF can be denoted as:

(1) $${\hat{C}}_{rf}^{B}\left(x\right)=\mathrm{Average}{\left\{{\hat{C}}_{b}\left(x\right)\right\}}_{1}^{B}$$

The 3RF voting-based output prediction is represented as follows:

(2) $$Y=\mathrm{Voting}({\hat{C}}_{rf_{i}}^{B}\left(x\right)),i=1\cdots 3$$

**Figure 2:**
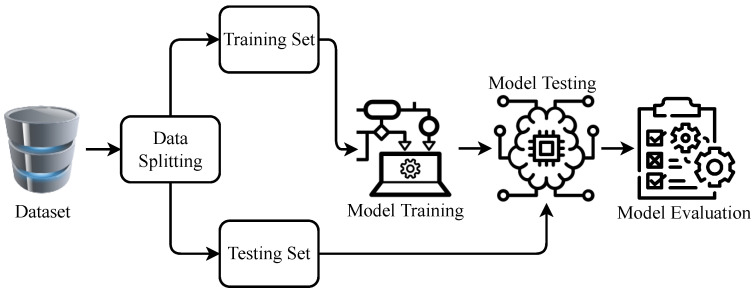
The architecture of the energy efficiency data processing pipeline.

The flow of the steps used to train and test the HL and DL datasets is depicted in Figure 2. The dataset is split into the training and testing dataset with proportions of 80% and 20%, respectively. The relation between the dataset features and the HL and CL output is analyzed to select the machine learning models. The feature versus HL and CL trends are also investigated to present the feature relation with the HL and CL output. The performance of the proposed model is evaluated in comparison to several well-known machine learning models.

### 3.2. Machine Learning Models

A brief overview of the employed machine learning models is described in this section. The grid search method is used to find the best hyperparameter setting. The model parameters are tuned between specific ranges to get the best results. Model hyperparameter setting and tuning range are shown in Table 2.

#### 3.2.1. K-Nearest Neighbors

KNN can be used for both classification and prediction. The prediction works based on feature similarity. The nearest neighbors were selected based on different distance measures. The uniform weight was used to assign equal weights to all neighbors. The number of neighbors was selected as three in our evaluation. The average of the nearest neighbor data values was assigned as the final predicted values. The Euclidean distance (E) was used to determine the nearest neighbors [20].

(3) $$E=\sqrt{\sum_{i=1}^{k}{\left(x_{i}-y_{i}\right)}^{2}}$$

where *k* denotes the number of neighbors, and $x_{i}$ and $y_{i}$ are the data points in $i^{th}$ dimension in the Equation (3).

#### 3.2.2. Linear Regression

LR works on the principle that the input independent variables are linearly related to the output dependent variable. The multiple linear regression is represented as [21]:

(4) $$y=\beta_{1}x_{1}+\beta_{2}x_{2}+\cdots +\beta_{r}x_{r}$$

where $x_{1}\cdots x_{r}$ are the input independent variables, *r* is the number of input features, and $\beta_{r}$ is the $r^{th}$ input feature coefficient in Equation (4).

In order to compensate for model overfitting, the error ε is modeled as a Gaussian distribution. The multiple linear regression in matrix form is represented as follows.

(5) $$y_{i}=x_{i}^{T}\beta +\varepsilon_{i}.$$

#### 3.2.3. Random Forest

RF is a family of decision tree-based machine learning algorithms. Ensemble learning is used to perform the classification and predictions. The bootstrapping RF is used to perform the predictions in this work. The bootstrapping method combines ensemble learning and the random selection of the decision trees to determine the prediction output as the average value of all the decision tree predictions.

Let b=1 and *B* be the number of decision trees; ${\hat{C}}_{b}\left(x\right)$ denotes the regression prediction value of the $b^{th}$ decision tree [19], then the regression prediction of the RF forest is defined as:

(6) $${\hat{C}}_{rf}^{B}\left(x\right)=\mathrm{Average}{\left\{{\hat{C}}_{b}\left(x\right)\right\}}_{1}^{B}$$

#### 3.2.4. General Additive Model

GAM is a slight variation of the linear regression model [22]. The linear form is replaced with the sum of the smooth functions in GAM. The function is represented as $f_{i}\left(x_{i}\right)$ for the $i^{th}$ input.This technique is helpful to detect the nonlinear covariate effects between input and output. The GAM is defined as follows:

(7) $$g\left(E\left(Y\right)\right)=\beta_{0}+f_{1}\left(x_{1}\right)+f_{2}\left(x_{2}\right)+\cdots +f_{r}\left(x_{r}\right).\;\;$$

where f(x) is the smooth function, *r* denotes the number of input features, E(Y) signifies the linear exponential output *Y* distribution, and g() is the link function in Equation (7). The link function can be selected as an identity or log function.

#### 3.2.5. Multilayer Perceptron

MLP is a global approximator and is well suited for mapping the nonlinear input–output combination. Typically, MLPs consist of three layers. The input layer feeds the input values to the neural network. The output layer performs the classification or prediction of the given problem. The hidden layer includes the neurons and supports the computations to process the input data and forwards the processed data as input to the output layer. The number of hidden layers can be arbitrary. The neuron processing unit is represented as follows [23].

(8) $$f\left(x\right)=\Phi \left(\sum_{i=1}^{m}w_{i}*x_{i}\right)+b$$

where *b* denotes the bias value, $W_{i}$ denotes the $i^{th}$ neuron weight, and $x_{i}$ denotes the input to the $i^{th}$ neuron unit. Φ is the nonlinear activation function and f(x) is the neuron processing unit output in the Equation (8).

### 3.3. Dataset Description

The heat and cool loading specifications must be determined when designing an energy-efficient building. The building feature characteristics will be used to determine the heat loading and cool loading. Thus, the building designer and designers expect to use the building features to estimate the heat loading and cool loading. Therefore, the target output parameters in the datasets are heat loading and cool loading for energy efficiency prediction. The dataset was collected from the University of California Irvine Machine learning laboratory.

The total surface (S) is determined as:

(9) S=Wall+2∗Floor

The Relative Compactness (RC) is measured as:

(10) $$RC=6*\frac{V^{23}}{S}$$

The energy efficiency dataset was acquired from the UCI repository, which was generated using Ecotect simulation software and presented by [18]. The dataset comprises 768 building samples, and each sample is represented with eight features. To generate 768 combinations, 12 building shapes are simulated in Ecotect. The 12 building shapes had the same volume (771.75 m^3^) and the features were varied. The variables orientation, glazing, and glazing distribution feature varied further to make the dataset. The orientation includes the building’s north, south, east, and west orientation. The glazing area or window-to-floor ratio varied from 10–40%. The four glazing area values taken were 0%, 10%, 25%, and 40% in the dataset. The glazing area distribution combinations included uniform, majority of the distribution in either north, south, east, or west. Overall, the total number of building combinations was (12 × 3 × 5 × 4) + (12 × 4) = 768. The detailed description of 8 features, the feature value ranges, and metrics are reported in Table 3.

### 3.4. Performance Metrics

The performance of the models was estimated using the metrics MAE, RMSE, MAPE, and the coefficient of determination $R^{2}$.

MAE measures the predicted value deviations from the original value. The MAE is the average difference between the predicted and original values. Given the CL and HL dataset output original value, $y_{i}$ and the dataset output predicted value was ${\hat{y}}_{i}$, and the total number of values i=0⋯N, then the MAE was calculated as:

(11) $$\mathrm{MAE}=\frac{1}{N}\sum_{i=1}^{N}\left|y_{i}-{\hat{y}}_{i}\right|$$

RMSE is the standard deviation of the prediction errors. The prediction error measures the distance of the prediction values from the regression line data values. RMSE is measured as:

(12) $$\mathrm{RMSE}=\sqrt{\frac{1}{N}\sum_{i=1}^{N}{\left(y_{i}-{\hat{y}}_{i}\right)}^{2}}$$

MAPE is the average of the predicted absolute percentage errors. The MAPE is obtained in percentages, and the sign of the predicted and original difference value does not matter. MAPE is measured as:

(13) $$\mathrm{MAPI}=100\%\times \sum_{i=1}^{N}\frac{|y_{i}-{\hat{y}}_{i}|}{y_{i}}$$

All three error metrics determine the deviation of the predicted value from the original value. Therefore, the lower the error metric value, the better the model performs.

The $R^{2}$ determines how well the model fits the data. The $R^{2}$ value lies between 0 and 1, and if the $R^{2}$ value for the model is close to 1, then the model performs well. Considering the mean of the dataset output predicted value is ${\hat{y}}_{i}$, the $R^{2}$ is defined as:

(14) $$R^{2}=1-\frac{\Sigma_{i=1}^{N}{\left(y_{i}-{\hat{y}}_{i}\right)}^{2}}{\Sigma_{i=1}^{N}{\left(y_{i}-{\bar{y}}_{i}\right)}^{2}}$$

## 4. Results and Discussion

In this section, a detailed description of the experimental results obtained on the cooling load and heading load datasets is provided. The experiments were run on a standalone Linux machine with a system configuration of 8 GB RAM and an eight-core processor. A notebook web application runs locally on the Linux machine to perform the experiments. The software packages scikit-learn installed, and the Python programming language was used to implement machine learning models.

### 4.1. Models’ Performance Analysis for Cooling Load

**Figure 3:**
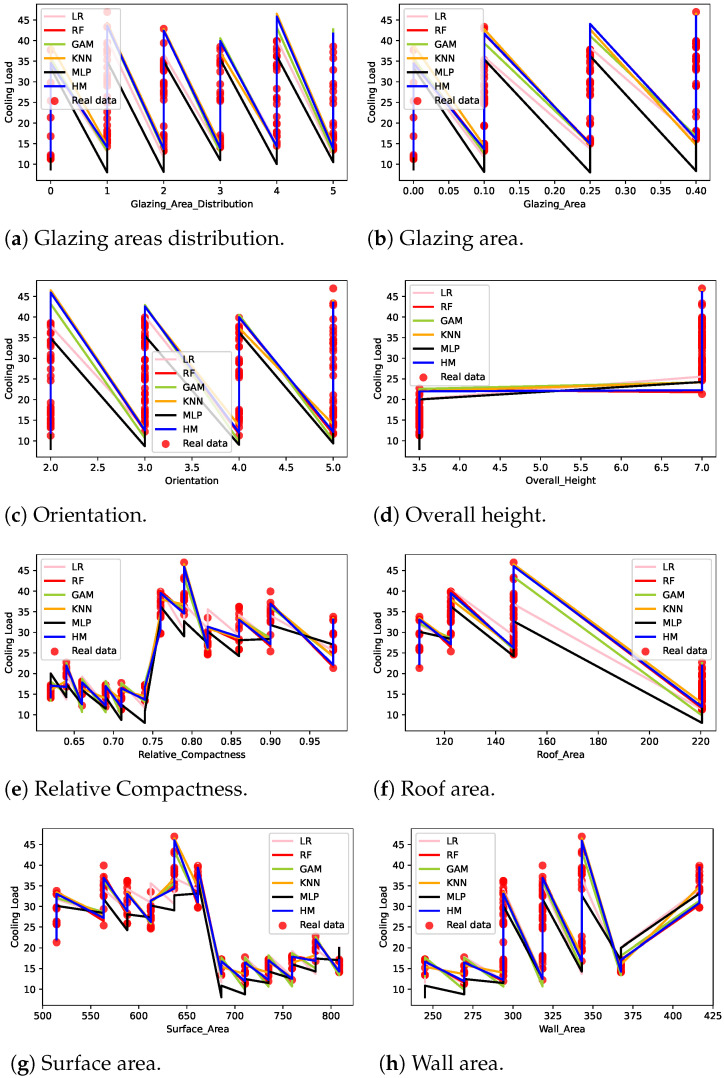
Cooling loading features from machine learning models.

Figure 3 shows the cooling load output value distribution for the energy efficiency dataset input features. The eight features, glazing area distribution, glazing area, orientation, overall height, relative compactness, roof area, surface area, and wall area mapping with cooling load values, were included in Figure 3. The circle indicates the real data value and the color lines represent cooling load values obtained when various machine learning techniques were applied for prediction. Results indicate that the real values highly deviate from the MLP line that represents the cooling load. On the other hand, the HM maps the input feature values more accurately with the cooling load data. Figure 3a–c look similar because the building features gazing area distribution, gazing area, and orientation have a similar dependency on the required cooling load values. Figure 3a shows that the cooling load varies significantly when the building height was either 3.5 or 7.0. When the building height was between 3.5 and 7.0, the cooling load was around 22. The other features, such as relative compactness, roof area, surface area, and wall area, had irregular dependency patterns with respect to the cooling load.

Table 4 displays the cooling load performance metrics for machine learning model regression analysis. It shows that the 3RF performed well compared to other machine learning models. The 3RF obtained the best $R^{2}$ of 0.971 for the cooling load. The 3RF error metrics MAE, MSE, and RMSE values were 2.875, 1.076, and 1.695, respectively. The MLP and LR did not perform well in measuring the cooling load accurately. The supervised models such as KNN, decision tree algorithms RF, 3RF, and GAM performed comparatively well with an $R^{2}$ value greater than 0.95. A similar pattern was found in the error metrics for the machine learning techniques in cooling load determination.

Table 5 lists the accuracy (mean $R^{2}$) and standard deviation of the machine learning techniques when testing the cooling load estimation. The 10-fold cross-validation was performed to split the dataset into training and testing datasets and measure the performance accuracy of the employed techniques. The performance accuracy of the machine learning models followed a similar trend of the $R^{2}$ metric value performance in Table 5. Here, 3RF obtained the best accuracy of 96% among the used models for cooling load estimation.

**Figure 4:**
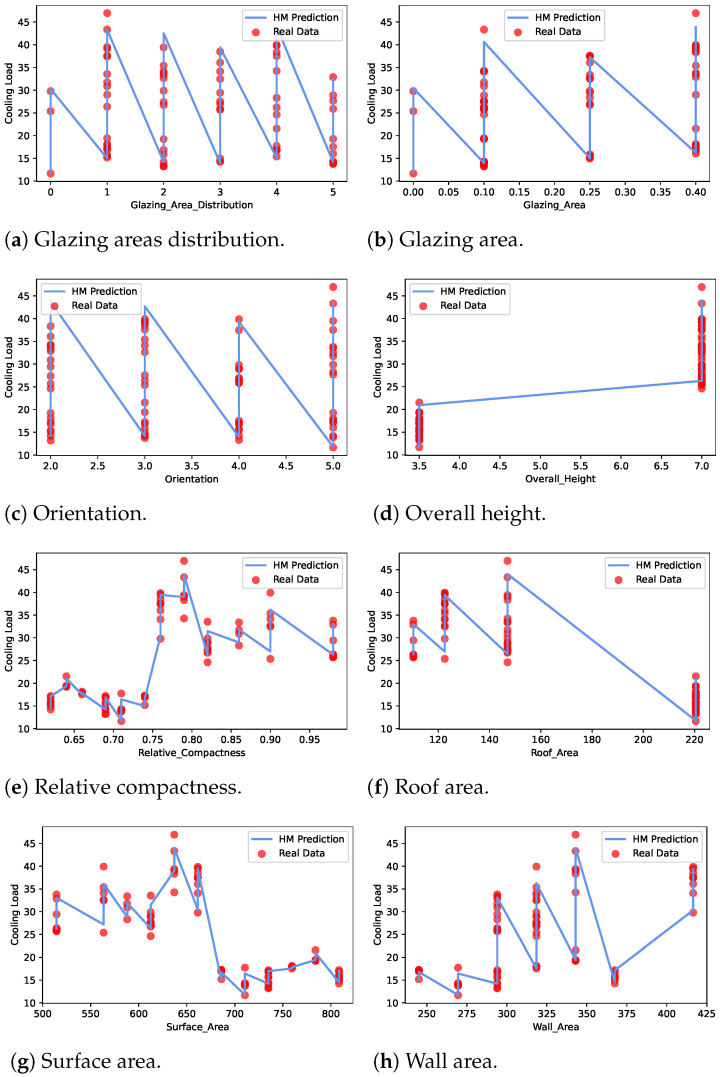
Cooling load features using the proposed 3RF model.

The best-performing 3RF model performance was analyzed with respect to the input features in cooling load estimation. Figure 4 presents the mapping of the feature data with the output cooling load when the 3RF model is used for estimation. The results indicate that most of the feature data samples match with the 3RF predicted cooling load output. Hence, the 3RF performance is better than the other ML models.

**Figure 5:**
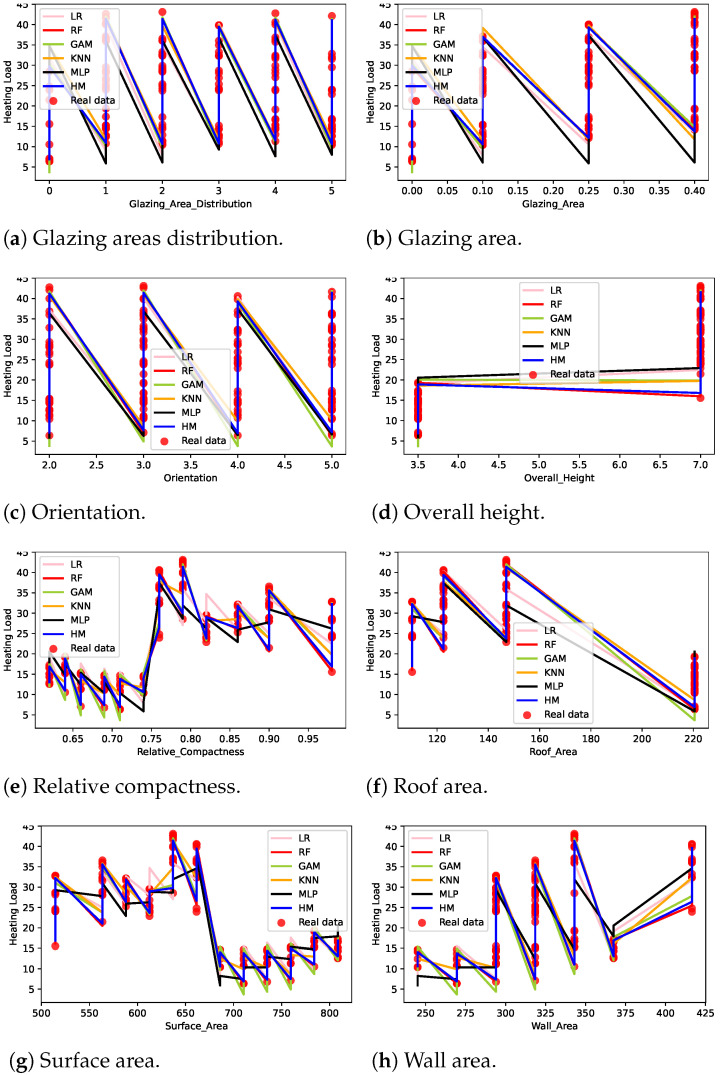
Heating load features using machine learning models.

The experiments were repeated for heating load performance estimation. As shown in Figure 5, the heating load mapping with respect to the input features is performed when the data is trained using machine learning models. The trends follow patterns similar to cooling load estimation. Figure 5 shows that the MLP model is missing some of the real data in the heating load estimation line for most of the input features. On the other hand, the 3RF matches the real data into the heating load prediction line. It means that 3RF performed well in accurately estimating the heat loading in a building.

### 4.2. Heat Loading Performance Analysis

Table 6 shows the performance of employed models in estimating a building’s heat loading. Results indicate that the 3RFF outperformed all other models in predicting heat loading. The 3RFF obtained the best performance in terms of $R^{2}$ with a 0.998 score and the lowest error metrics value. The RF and GAM comparatively performed well with more than 0.99 $R^{2}$ value. The MLP and LR were the least-performing models to estimate heat loading. Overall, the results indicate that decision tree-based techniques work well to comprehend the energy efficiency input features and correctly estimate the heat and cool loading values.

Table 7 displays the performance of machine learning models for heating load prediction problems. The 10-fold cross-validation method is used to split the datasets into train and test sets. Results show that the RF achieved the best performance accuracy on the heat loading estimation. A similar trend in Table 6 is observed for the machine learning performance accuracy results. The decision tree algorithms performed well to obtain the best accuracy for the heating load case. The standard deviation for each model indicates that the accuracy can be slightly varied, and the obtained accuracy results may not be the same when repeated cross-validation experiments are carried out. Table 8 shows the per epochs score for 3RF in both cooling and heating cases.

Table 8 shows the fold-wise cross-validation results using the best performing 3RF model. Fold-wide results are provided to analyze the fold-wise variance in the prediction accuracy of the 3RF model. It can be observed that, except for the first fold, the results are consistent with small variations in the prediction accuracy of building heating and cooling.

**Figure 6:**
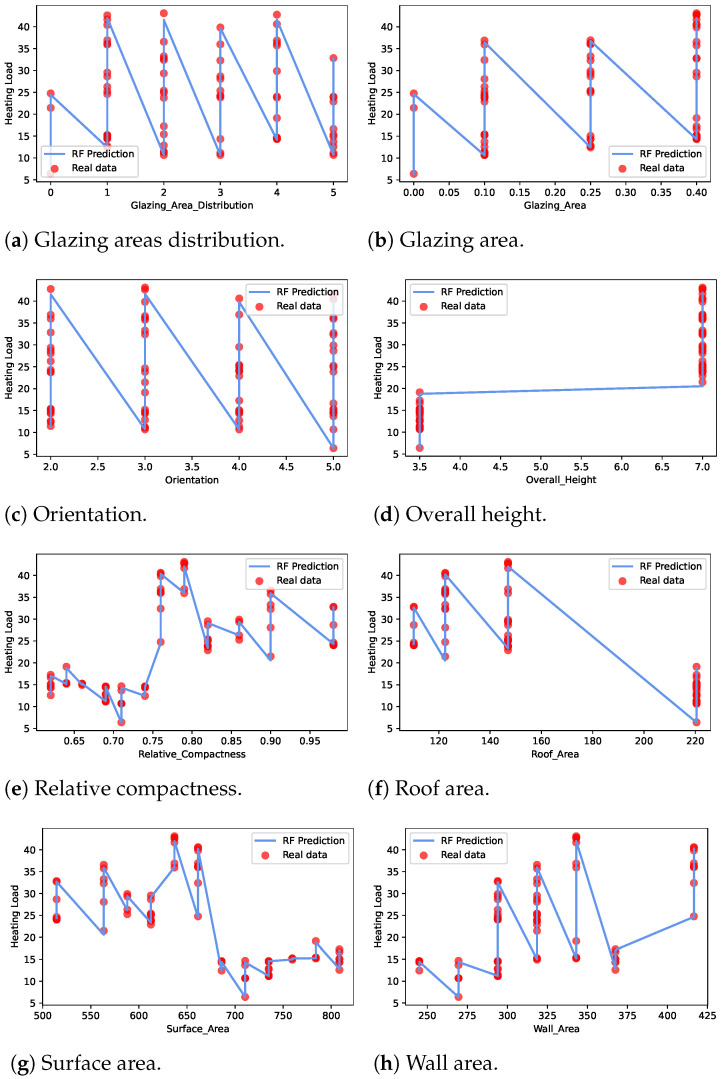
Heating load prediction using best performing RF features.

The best performing RF model for heat loading prediction per the energy efficiency dataset features is shown in Figure 6. The subfigures show the real data points, and the RF predicted heat loading estimation line graph maps for all the feature data. Overall, the features such as grazing area distribution, glazing area, orientation, relative compactness, surface area, and wall area greatly influence the heat loading required in a building. Therefore, selecting the optimal building parameters is crucial for building energy efficiency.

**Figure 7:**
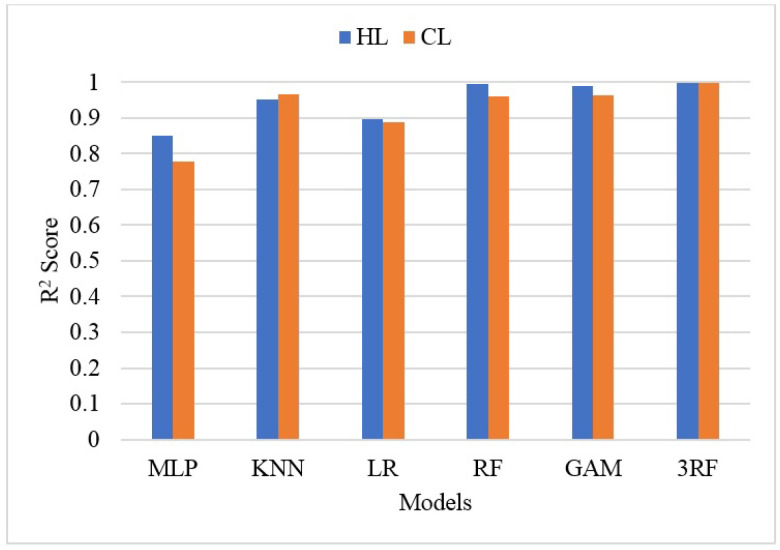
Performance comparison of machine learning models.

Figure 7 depicts the performance comparison of both HL and CL prediction using the machine learning techniques KNN, LR, RF, and 3RFF. The results indicate that the proposed 3RFF performed better than the other ML models for HL and CL prediction. The 3RFF obtained an accuracy of 0.95 for both HL and CL prediction. The SD of the proposed HL accuracy prediction is higher than the CL accuracy prediction. The KNN is the least performed model for both HL and DL prediction. The MLP model was the least performed model among the selected HL and CL evaluation models. The ensemble models such as RF performed better than the linear regression model for energy efficiency problems. Our findings match the prior works, which showed that the ensemble learning models obtained the best HL and CL performance prediction. Overall, the performance accuracy results in Figure 7 proved that the proposed model performed well and outperformed the employed models in this work.

Besides using the 3RF model, different ensemble schemes were used to achieve the optimal results such as different combinations of RF, LR, and KNN. For the most part, the existing literature uses only two models for ensemble models. Keeping this in mind, three models were used for the ensemble. For this purpose, the 3LR ensemble was also tried but the performance of the model was not as good as using the 3RF. In addition, 4RF and 5RF were also implemented but there was no improvement in results after 3RF. To avoid computational costs for 4RF and 5RF, a 3RF ensemble was used. Table 9 is provided to show the performance of 2RF, 3RF, 4RF, and 5RF regarding the prediction accuracy and computational complexity. It can be observed that increasing the RF models from 3 to 4 and 5 does not add any value regarding accuracy. Instead, the computational time increased. Thus, the best results were obtained using 3RF for building heating and cooling prediction.

### 4.3. Performance of Deep Learning Models

State-of-the-art deep learning algorithms such as convolution neural network (CNN), long short-term memory (LSTM), and the combination of CNN and LSTM have been considered to evaluate the HL and CL performance.

The CNN comprises the embedding layer, 1D convolution layer, 1D max-pooling layer, flatten layer, and dense layer. The ReLu activation function is used to perform the convolution. A pool size value of four was also considered to downsample the feature map. The ’Adam’ optimizer and MAE loss function was used to train the CNN models. The dataset was split as training and testing data with 80% and 20% ratios. The batch size of 128 and 2000 epochs were selected to train the CNN models. Complete details of the CNN hyperparameters considered for HL and CL evaluation are given in Table 10.

LSTM is a type of recurrent neural network (RNN). A 100-unit LSTM layer was used in this work to design LSTM. Dropout and dense layers were also included to train the LSTM model for HL and CL prediction. The same CNN batch size, epochs, and optimizer, loss function parameters were considered to train the LSTM model.

The CNN and LSTM models were combined to evaluate the HL and CL performance. The convolution, max pooling, and LSTM models were included in the CNN-LSTM model. The set of hyperparameters used in CNN and LSTM was also used in the CNN-LSTM. Table 10 displays all the hyperparameters used for CNN, LSTM, and CNN-LSTM models.

#### 4.3.1. Cooling Load Prediction using Deep Learning

**Figure 8:**
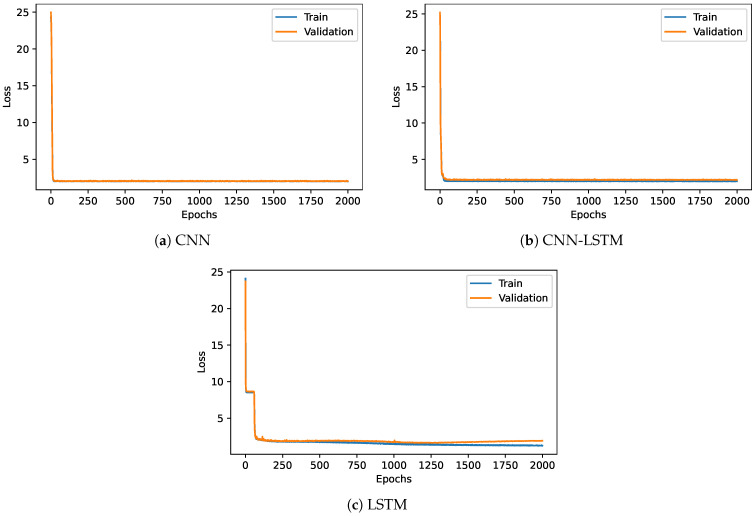
Training and validation loss for deep learning models for cooling load.

The cooling load estimation was also performed using deep learning techniques such as CNN, LSTM, and CNN-LSTM. Figure 8 displays the loss of the three models when the epochs vary from 0 to 2000 for both the validation and train dataset. The model loss was promptly reduced from 25% to 2% when the first epoch was completed in both CNN and CNN-LSTM models. The model loss maintains 2% until the completion of the epoch 2000. The model loss was drastically reduced during the first few epochs in the LSTM model and then settled to 3% loss when the epoch completed 60 epochs. The model loss maintained a constant 3% from epoch 60 to 2000 epochs for both the train and validation dataset.

Table 11 displays the performance metrics of the three deep learning models when tested on the cooling load estimation. LSTM performed well compared to CNN and CNN-LSTM models with an $R^{2}$ value of 0.933. The combination of LSTM and CNN degraded the performance compared to the LSTM and CNN alone. The error metrics followed the inverse trend of the $R^{2}$ on all three models. The best performing deep learning model LSTM obtained the MAE, MSE, and RMSE values of 5.82, 1.92, and 2.41, respectively. Overall, the proposed 3RF obtained the best performance in cooling load estimation for building energy efficiency.

#### 4.3.2. Heat Load Prediction using Deep Learning

**Figure 9:**
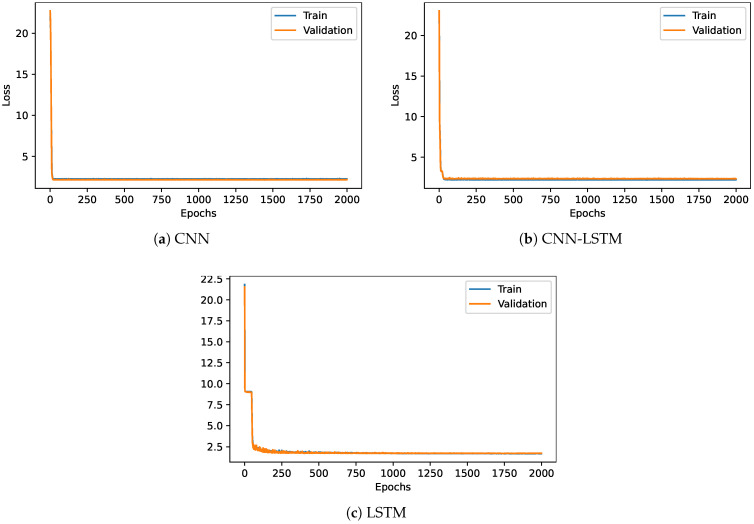
Models training and validation loss for heating loading.

Deep learning models CNN, LSTM, and CNN-LSTM are used for the estimation of the heating load. Figure 9 shows the model loss of the three DL models when the epochs vary from 0 to 2000 for both train and validation datasets. The model loss for both the cool loading and heat loading estimation followed a similar pattern. When CNN was trained and validated, the model loss abruptly dropped to 2% when epoch one was completed. On the other hand, the LSTM model needed at least 60 epochs to reach the 2% model loss. The CNN-LSTM and CNN models did not change the model loss value from epochs 30 to 2000. Further, CNN obtained the best model accuracy within the first few epochs compared to the LSTM.

Table 12 presents the performance of deep learning models for the heating load case. When the error metrics and $R^{2}$ values were considered, the LSTM model performed better than the CNN and CNN-LSTM. The LSTM obtained the $R^{2}$ value of 0.95 and the RMSE value of 2.22. The CNN produced the $R^{2}$ value of 0.90, slightly less than the LSTM. The CNN-LSTM had the worst performance and obtained an $R^{2}$ value below 0.90. The result indicates that the combination of CNN and LSTM does not perform well for regression problems, where the output value varies based on the number of input features.

Results of 10-fold cross-validation are also added for deep learning models. Results indicate that LSTM achieved better results for both heating load and cooling load prediction as it achieved 0.90 for heating load prediction and 0.88 for cooling load prediction, as shown in Table 13. CNN and CNN-LSTM perform marginally lower than LSTM.

### 4.4. Performance Comparison with State-of-the-Art Studies

The proposed 3RF-based building HL and CL prediction are compared with the prior works, which utilized the same dataset for experiments. For the HL case, the ensemble learning models obtained good performances with $R^{2}$ values ranging from 0.98 to 0.998. The prior works [24,25,26] leveraged the RF or ensemble learning models and obtained a 0.998 $R^{2}$. Ref. [6] utilized the component-based machine learning techniques to predict the HL. The performance of the component-based work is much lower than the best HL prediction performance. The proposed 3RF model obtained the best performances as compared to existing ensemble models with an $R^{2}$ value of 0.999. The proposed model is also efficient in terms of computational cost, as it takes 0.83 seconds(s) for the cooling prediction case for model training and testing, and for the heating case it takes only 0.61 s.

Existing work [24] on CL performance results reported an $R^{2}$ value of 0.986 when the ensemble model was used for evaluation. As shown in Table 14, the proposed method obtained an $R^{2}$ value of 0.997. It indicates that the proposed model performed well to predict the CL. It is also observed that for CL prediction, the proposed model performed better than the RF model proposed in [26] with an $R^{2}$ value of 0.991 and the XGBoost model proposed in [10] with an $R^{2}$ value of 0.94. The multi-layer perceptron model proposed in [17] obtained the CL prediction with a 0.9222 $R^{2}$, which is far less than the current work. It is also important to note that deep learning models require higher computational resources and require a large amount of data to train the model.

### 4.5. Discussion and Limitations

The building energy efficiency problem is a significant problem for sustainable and smart homes. The accurate prediction of the cooling and heating load for the building structure helps the designers to design energy-saving building structures for homeowners and indirectly helps society by saving energy consumption. This study extensively analyzes the building features, cooling load, and heating load dependency on the features using data analytics models. The RF-based decision tree models estimate the cooling and heating load most accurately per the building features. The relative compactness, surface area, and wall area play a significant role in selecting the appropriate cooling and heating load for a building. However, the dataset used in this study is limited to 768 building designs and the corresponding cooling and heating load values. For further validation, a larger dataset is needed to test the regression performance of heating and cooling load estimation. The deep learning models CNN and LSTM did not perform well for energy load prediction. Further experiments using deep learning models with a larger dataset are intended.

## 5. Conclusions

Energy consumption prediction is an important research problem for sustainable homes and modern smart cities. Cooling and heating load prediction helps designers make energy-sustaining architectures. Modern buildings and smart cities employ a large number of IoT devices that generate large amounts of data regularly. Data-driven approaches leverage this data for cooling and heating load prediction. This study proposed a novel ensemble model, 3RF, to predict buildings’ cooling and heating profiles using different features such as glazing area, orientation, height, relative compactness, roof area, surface area, and wall area. Prediction within the context of various features indicates that relative compactness, surface area, and wall area play a significant role in selecting the appropriate cooling and heating load for a building. Experimental results using the proposed model suggest that the model achieves 0.999 $R^{2}$ for heating load prediction and 0.997 $R^{2}$ for cooling load prediction. The model’s performance is superior to both the employed and existing state-of-the-art models. Precise prediction of energy requirements of modern buildings can be very important for energy efficiency and sustainability. Moreover, such predictions can be used for a large number of applications such as energy monitoring, real-time energy planning, and identifying high energy-consuming targets, which can help optimize energy efficiency. The current dataset consists of energy profiles for 768 buildings and seems insufficient for deep learning models. Enlarging the dataset and improving the performance of deep learning models is also intended as future work.

## Figures and Tables

**Table 1:** Comparative analysis of discussed works on heating and cooling load prediction.

Ref.	Year	Dataset	Model	Target	Evaluation Metrics	Results
[5]	2018	UCI	ELM, OSELM	HL & CL	MAE, Prediction Time(PT)	RBF—HL: MAE 0.0456 PT 0.0389; CL: MAE 0.0358 PT 0.0348
[6]	2018	Energy Plus Simulation	ANN	HL & CL	$R^{2}$, Maximum Deviation	ANN—Maximum Deviation HL: 3.7% CL: 3.9%; $R^{2}$ HL:0.974 CL:0.999
[8]	2018	Energy Plus Simulation	DL	HL & CL	$R^{2}$	$R^{2}$—CL:0.983; HL:0.848
[9]	2019	UCI	XGBoost	HL & CL	RMSE, $R^{2}$, MAE, MAPE	XGBoost—HL: RMSE(kW) 0.265, $R^{2}$ 0.9993, MAE(kW) 0.175, MAPE(%) 0.193; CL: RMSE(kW) 0.307, $R^{2}$ 0.461, MAE(kW) 1.197, MAPE(%) 0.998
[10]	2019	Synthetic Dataset	XGBoost, ANN	HL & CL	RMSE, $R^{2}$	XGBoost—CL: RMSE 2.95, $R^{2}$ 0.98; HL: RMSE 3.90, $R^{2}$ 0.95
[7]	2019	UCI	Octahedric Regression(OR)	HL& CL	MAE, MSE, MAPE	OR—HL: MAE 0.945, MSE 2.289, MAPE 4.182, CL: MAE 1.113, MSE 2.731, MAPE 4.554
[11]	2020	Energy Plus Simulation	Multi-objective optimisation	HL & CL	RMSE, MAE, $R^{2}$	HL: RMSE 10.25, MAE 2.54, $R^{2}$ 0.992 CL: RMSE 6.63, MAE 2.36, $R^{2}$ 0.993
[12]	2020	UCI	DNN	HL & CL	NMAE, MAE, NRMSE, RMSE, $R^{2}$	DNN—HL: NMAE 0.018, MAE 0.2, NRMSE 0.025, RMSE 0.263, $R^{2}$ 1; CL: NMAE 0.03, MAE 0.485, NRMSE 0.039, RMSE 0.69, $R^{2}$ 0.994
[13]	2020	Heat Exchange Stations in Anyang City data	Temporal convolutional neural network (TCN)	HL	MAE, RMSE, MAPE, Accuracy	TCN—MAE 0.102, RMSE 0.129, MAPE 0.021, Accuracy 0.979
[14]	2020	UCI	Gated Recurrent Unit(GRU)	HL & CL	MAE, MSE, RMSE, MAPE	GRU—HL: MAE 1.3691, MSE 0.7215, RMSE 0.8494, MAPE 0.9315; CL: MAE 1.4027, MSE 0.9791, RMSE 0.9894, MAPE 1.0132
[15]	2020	Office Building in Tianjin China dataset	K-means clustering, Discrete Wavelet Transform(DWT)	CL	$R^{2}$, CV-RMSE	$R^{2}$ 99.8%, CV-RMSE 1.5%,
[16]	2022	UK Ministry of Housing dataset	DT, SVM, Gradient Boosting(GB) and all ML	Energy Efficiency	Accuracy	GB—Accuracy 0.67
[17]	2022	UCI	Shuffled complex evolution(SCE)-multi-layer perception(MLP)	CL	RMSE, MAE, $R^{2}$	SCE-MLP—RMSE 2.5943, MAE 0.8124, $R^{2}$ 0.9227

**Table 2:** Machine learning model hyperparameter settings.

Model	Hyperparameters	Tuning Range
MLP	random_state=1, max_iter=500	random_state={1 to 10}, max_iter={100 to 1000}
KNN	n_neighbour=3, weights=’uniform’	n_neighbour={1 to 5}, weights=’uniform’
LR	Default	Default
RF	n_estimators=300, max_depth=10	n_estimators={50 to 500}, max_depth={2 to 50}
3RF	RF+RF+RF	2RF, 3RF, 4RF, 5RF

**Table 3:** Description of dataset features.

Feature	Combinations	Value Range	Unit	Description
**Input**				
RelativeCompactness	12	0.68–0.98	-	The volume to surface ratio is compared the most compact shape with same volume
SurfaceArea	12	514–808	m^2^	The total area occupied by the building
WallArea	7	245–416	m^2^	Total area of an exterior building wall including all openings
RoofArea	4	110–220	m^2^	the surface of the roof of the building
OverallHeight	2	3.5–7	m	Overall height from the lowest point of conditioned space to the highest point
Orientation	4	2–5	-	Orientation decides which direction the building faces
GlazingArea	4	0–0.4	m^2^	The total area occupied by windows in a building
GlazingAreaDistribution	6	0–5	-	The direction of the glazing area covered in the building
**Output**				
Heating Load	-	6–43	KWh/m^2^	Amount of heat added in an area to maintain the temperature within acceptable range
CoolingLoad	-	10–48	KWh/m^2^	The amount of latent and sensible heat removed from the required area to maintain the acceptable temperature

**Table 4:** Cooling load prediction using machine learning models.

Model	MAE	MSE	RMSE	$R^{2}$
MLP	21.835	3.475	4.672	0.777
KNN	2.946	1.351	1.716	0.966
LR	9.652	2.220	3.106	0.887
RF	3.424	1.101	1.850	0.959
GAM	3.471	1.385	1.863	0.964
3RF	0.515	0.526	0.675	0.997

**Table 5:** Cooling load prediction results using 10-fold cross-validation for machine learning models.

Model	Mean $R^{2}$	Standard Deviation
MLP	0.76	+/−0.12
KNN	0.91	+/−0.08
LR	0.91	+/−0.08
RF	0.91	+/−0.08
GAM	0.90	+/−0.08
3RF	0.96	+/−0.03

**Table 6:** Heating load prediction performance using machine learning models.

Model	MAE	MSE	RMSE	$R^{2}$
MLP	14.241	2.885	3.773	0.849
KNN	5.185	1.729	2.277	0.950
LR	10.260	2.353	3.203	0.896
RF	0.369	0.372	0.607	0.996
GAM	1.060	0.749	1.029	0.990
3RF	0.521	0.548	0.722	0.998

**Table 7:** Heating load results using 10-fold cross-validation for machine learning models.

Model	Mean $R^{2}$	Standard Deviation
MLP	0.70	+/−0.15
KNN	0.85	+/−0.08
LR	0.89	+/−0.05
RF	0.92	+/−0.08
GAM	0.91	+/−0.08
3RF	0.95	+/−0.08

**Table 8:** Fold-wise cross-validation accuracy for 3RF model.

Fold	Heating Load	Cooling Load
1	0.724	0.862
2	0.975	0.982
3	0.976	0.958
4	0.973	0.976
5	0.981	0.989
6	0.974	0.966
7	0.978	0.982
8	0.976	0.968
9	0.973	0.956
10	0.985	0.975
Mean	0.95 +/− 0.08	0.96 +/− 0.03

**Table 9:** Accuracy and computational cost (time) for different variants of RF.

Model	Heating Load		Cooling Load	
	$R^{2}$ Score	Time (s)	$R^{2}$ Score	Time (s)
2RF	0.996	0.59	0.996	0.747
3RF	0.998	0.61	0.997	0.83
4RF	0.997	1.34	0.997	1.97
5RF	0.997	1.67	0.997	1.87

**Table 10:** Hyperparameters used for the deep learning models for HL and CL prediction.

Parameters	CNN	LSTM	CNN-LSTM
Conv1D	-	1	1
MaxPooling1D	-	Yes	Yes
Poolsize	-	4	4
Dense	1	1	1
Dropout	0.2	-	-
Activation	ReLU	ReLU	ReLU
Batchsize	128	128	64
Optimizer	Adam	Adam	Adam
Loss	MAE	MAE	MAE
Number of Units	128	128	128
Epochs	2000	2000	2000
Flatten	Yes	Yes	No

**Table 11:** Error statistics of deep learning models for cooling load.

Model	MAE	MSE	RMSE	$R^{2}$
LSTM	5.82	1.92	2.41	0.93
CNN	7.23	2.03	2.69	0.92
CNN-LSTM	8.33	2.18	2.89	0.90

**Table 12:** Performance of deep learning models for the heating load.

Model	MAE	MSE	RMSE	$R^{2}$
LSTM	4.95	1.73	2.22	0.95
CNN	9.23	2.17	3.04	0.90
CNN-LSTM	11.21	2.33	3.35	0.87

**Table 13:** Results using 10-fold cross-validation for deep learning models.

Model	Heating Load		Cooling load	
	Mean $R^{2}$	Standard Deviation	Mean $R^{2}$	Standard Deviation
LSTM	0.90	+/−0.08	0.88	+/−0.11
CNN	0.89	+/−0.14	0.85	+/−0.12
CNN-LSTM	0.89	+/−0.05	0.87	+/−0.09

**Table 14:** Comparison with state-of-the-art approaches (metric: $R^{2}$).

Authors	Year	Model	HL	CL
[24]	2014	Ensemble model	0.998	0.986
[25]	2014	Ensemble model	0.998	0.990
[26]	2017	RF	0.998	0.991
[6]	2018	Component based	0.848	0.983
[10]	2019	XGBoost	0.98	0.94
[17]	2020	SCE-MLP	-	0.922
Current study	2022	3RF	0.999	0.997

## Data Availability

The dataset link: https://archive.ics.uci.edu/ml/datasets/energy+efficiency (accessed on 9 September 2022).
